# Endothelial cells release cardioprotective exosomes that may contribute to ischaemic preconditioning

**DOI:** 10.1038/s41598-018-34357-z

**Published:** 2018-10-26

**Authors:** Sean M. Davidson, Jaime A. Riquelme, Ying Zheng, Jose M. Vicencio, Sergio Lavandero, Derek M. Yellon

**Affiliations:** 1 The Hatter Cardiovascular Institute, University College London, London, United Kingdom; 2 Advanced Center for Chronic Diseases, Facultad de Ciencias Químicas y Farmacéuticas, Universidad de Chile, 8380494 Santiago, Chile; 3 Cancer Institute, University College London, London, UK

## Abstract

Extracellular vesicles (EVs) such as exosomes are nano-sized vesicles that carry proteins and miRNAs and can transmit signals between cells. We hypothesized that exosomes from endothelial cells can transmit protective signals to cardiomyocytes. Co-culture of primary adult rat cardiomyocytes with normoxic HUVEC cells separated by a cell-impermeable membrane reduced the percentage of cardiomyocyte death following simulated ischaemia and reperfusion (sIR) from 80 ± 11% to 51 ± 4% (P < 0.05; N = 5). When EVs were removed from the HUVEC-conditioned medium it was no longer protective. Exosomes were purified from HUVEC-conditioned medium using differential centrifugation and characterized by nanoparticle tracking analysis, electron microscopy, and flow cytometry. Pre-incubation of cardiomyocytes with HUVEC exosomes reduced the percentage of cell death after sIR from 88 ± 4% to 55 ± 3% (P < 0.05; N = 3). This protection required ERK1/2 activity as it was prevented by inhibitors PD98059 and U0126. Ischaemic preconditioning caused about ~3-fold higher rate of exosome production from HUVEC and from isolated, perfused rat hearts. This increase resulted in significantly greater protection against sIR in cardiomyocytes. In conclusion, exosomes released from endothelial cells can confer resistance to sIR injury in cardiomyocytes via the activation of the ERK1/2 MAPK signalling pathway, and may contribute to IPC.

## Introduction

Ischaemia and reperfusion injury (IRI) is a major contributing factor to the death of cardiomyocytes that occurs during myocardial infarction^1,2^. Ischaemic preconditioning (IPC), consisting of short, non-lethal periods of ischaemia and reperfusion, has been known for many years to be one of the most powerful ways to protect the heart from subsequent IRI^2–4^. The intracellular signalling pathway required for IPC requires the activation of PI3Kinase/Akt or MAPK/ERK1/2, referred to as the reperfusion injury salvage kinase (RISK) pathway^5^. IPC can also protect the heart when it is applied to an organ or limb remote from the heart, in what is known as remote IPC (RIPC)^6,7^.

It has recently been suggested that exosomes might be involved in the mechanism of IPC and RIPC^8,9^. Exosomes are nano-sized extracellular vesicle (EVs) released by most cell types^10–13^. Unlike larger EVs such as microvesicles, which are released by shedding from the plasma membrane, exosomes are released via fusion of multivesicular bodies with the plasma membrane. Interest in exosomes has increased greatly since they were shown to be able to induce acute cardioprotection^14^. In addition, exosome administration results in long-term improvement in ventricular function via various pathways including the stimulation of angiogenesis, immunosuppression, and potentially the activation of regenerative pathways^12,13^. Various types of stem cells have been investigated as potential sources of cardioprotective exosomes, and paracrine signalling via exosomes is now believed to mediate much of the cardiovascular benefit that has been seen after stem cell injection^15^. As mentioned, however, large numbers of exosomes are continually released into the circulation by different cell types including platelets, erythrocytes, leukocytes and endothelial cells, and these may also contribute to cardiovascular protection. We showed previously that exosomes purified from plasma are cardioprotective^16^, although, interestingly, this protection was lost when the exosomes were isolated from rats or humans with type II diabetes^17^.

The internal lamina of most vessels of the cardiovascular system is lined by a thin layer of endothelial cells, which help to regulate vessel tone in addition to providing trophic support via signalling to the underlying parenchyma^18^. In the heart, the endothelium is non-fenestrated, and performs an additional, important barrier function between the blood and the cardiomyocytes. It is increasingly recognized that endothelial cells function as more than simple barriers in the cardiac vasculature, and can also actively collaborate with the underlying cardiomyocytes and modulate cardiac function (reviewed in^18,19^). We used a co-culture model with both human umbilical vein endothelial cells (HUVEC) and primary adult rat cardiomyocytes separated by a cell-impermeable membrane, to investigate whether endothelial cells release exosomes that can stimulate cardioprotection in recipient cardiomyocytes, whether IPC increases the release of these nano-sized vesicles, and whether these might contribute to preconditioning.

## Material and Methods

### Ethical approval

All procedures contained within the application have been reviewed by the institutional veterinary surgeon Olga Woolmer (2017). The experimental protocols were approved by the Animal Welfare and Ethical Review Body (AWERB). The experiments are conducted within the terms of the Animals (Scientific Procedures) Act 1986, under Project Licence number PPL 70/8556, (“Protection of the Ischaemic and Reperfused Myocardium”) issued to Prof. Derek Yellon in 2015. All animals received humane care in accordance with the United Kingdom Home Office Guide on the Operation of Animal (Scientific Procedures) Act of 1986. The investigation conforms to the guidelines from Directive 2010/63/EU of the European Parliament on the protection of animals used for scientific purposes or the NIH guidelines.

### Primary cardiomyocyte isolation

Male Sprague Dawley Rats (between 200–300 g) were anesthetized with 200 mg/kg i.p. sodium pentobarbital by intraperitoneal injection. Cardiomyocytes were isolated from isolated, perfused hearts using a standard method of collagenase II digestion, and plated in laminin-coated dishes. Cells were subject to hypoxia and reoxygenation (H/R) by replacing the medium with hypoxic buffer simulating ischaemia, containing 128 mM NaCl, 2.2 mM NaHCO_3_, 14.8 mM KCl, 1.2 mM MgSO_4_, 1.2 mM K_2_HPO_4_, 1 mM CaCl2, 10 mM Na.lactate (pH 6.4) and placing the cells into a hypoxic chamber (<0.1 mmHg), in which the air is replaced 95% N_2_/5% CO_2_ for 2.5 h, after which cells were placed into a standard incubator in normal medium for 30 min. Control cells were incubated in normoxic buffer composed of 118 mM NaCl, 22 mM NaHCO_3_, 2.6 mM KCl, 1.2 mM MgSO_4_, 1.2 mM K_2_HPO_4_, 1 mM CaCl_2_, 10 mM glucose (pH 7.4 gassed with 95% O_2_/5% CO_2_).

### Cell culture

HUVECs were cultured in EGM-2 BulletKit (CC-3156 & CC-4176) from Lonza. Cells were routinely passaged with Accutase from Sigma. Experiments were performed using HUVEC cells between approximately passage 3 and 9. Cells were discarded after passage 12. For co-culture experiments, 80,000 HUVEC cells were seeded into each of Millicell 6-well plate inserts with 0.4 μm pores (Millipore) in normal culture medium. Cells were allowed to attach for 24 h. The medium was then either replaced with normoxic buffer (for control HUVEC), or with hypoxic buffer and the cells subject to 30 min hypoxia to precondition HUVEC. Both control and preconditioned HUVEC were then replenished with normoxic buffer and the inserts were placed into the wells of 6 well plates previously plated with freshly isolated cardiomyocytes in normoxic buffer. These were left for 45 min to allow transfer of cardioprotective factors from HUVEC to cardiomyocytes. The inserts were then removed, and the cardiomyocytes were subject to *in vitro* 2.5 h hypoxia in a buffer simulating ischaemia followed by 30 min reoxygenation in normoxic buffer. The population of dead cardiomyocytes was detected by staining with 1 mg/ml vital dye propidium iodide (PI) and imaging on a fluorescent microscope (Nikon). For time-course experiments, 200,000 cells were seeded into each well of duplicate 6-well plates and allowed to attach for 24 hours. The medium was either replaced with normoxic buffer or replaced with hypoxic buffer and placed in a hypoxic chamber for 30 min. After 30 min, the buffer was replaced with fresh normoxic buffer, and 1 ml of supernatant was removed for analysis after a further 30 min, 60 min, and 90 min. The supernatant was centrifuged at 300 × g for 10 min, and then 10, 000 × g for 30 min to remove larger particles and cellular debris. The pellets were discarded and the supernatants were analysed using NTA as described below.

### Exosome preparation

HUVECs were grown to approximately 90% confluency. To obtained IPC exosomes, HUVECs were subject to simulated ischaemia and reoxygenation by replacing the medium with an ischaemic buffer and placing them into a hypoxic chamber (<0.1 mmHg) for 30 min, whereas for control exosomes, the medium was replaced with a normoxic buffer for 30 min, then both the hypoxic and normoxic buffer was replaced with exosome-free culture medium. The cells were returned to the incubator for 24 h, and the supernatant was harvested. Exosomes were prepared using the differential centrifugation method described by Thery *et al*. as follows: 300 × g for 10 minutes at 4 °C to get rid of dead cells and debris, then 10,000xg for 30 minutes at 4 °C to remove large microvesicles, then twice at 100,000 × g for 120 minutes at 4 °C with a SW-41 rotor, including a PBS wash step^20^.

### Exosome Electron Microscopy and Nanoparticle tracking analysis

Electron Microscopy was performed on a Joel 1010 transition electron microscope (Joel Ltd, Warwickshire, UK) after a standard staining procedure with 0.5% uranyl acetate. Exosome preparations were diluted in PBS and examined using a Nanosight LM10-HS (Nanosight Ltd) equipped with a 405 nm laser beam and using constant flow injection. In this technique, the paths of particles undergoing Brownian motion is tracked from the scattered light. Nanoparticle tracking analysis (NTA) was used to calculate the concentration and size of isolated particles. The mean squared displacement was determined for each particle, with the diffusion coefficient and sphere-equivalent hydrodynamic radius then determined using the Stokes-Einstein equation. Results are displayed as a particle size distribution. Nanoparticle Tracking Analysis (NTA) 2.3 Analytical software was utilised with detection threshold set at 2–5. 5 videos of 30 s were recorded for each sample and the data from at least 5,000 individual particle tracks was analysed per sample.

### Flow cytometry

An indirect flow cytometric method was used to analyse the presence of exosomal marker CD63, since exosomes are too small for direct analysis by flow cytometry. Exosomes were bound to 4 μm aldehyde/sulphate latex microspheres, blocked, then incubated with CD63 primary antibody, and fluorescent secondary antibodies^16^. Briefly, macromolecular IgG complexes were cleared from 2 × 10^9^ exosomes (quantified by NTA) by an immunoprecipitation pre-clearing protocol using G protein Sepharose (Protein G Sepharose, GE Healthcare). Exosomes were then bound to 5 μl of aldehyde/sulphate latex microspheres (4% w/v, 4 μm Molecular Probes), in 1 ml of sterile PBS with agitation at 4 °C. Exosome-coated microspheres were blocked with 3% fatty acid-free BSA for 1 h at 4 °C, then incubated with isotype control or CD63 (clone H193. Secondary antibodies Alexa Fluor488 were used. For the analysis of microspheres, we used singlet-gated events on SSC/pulse-width. All samples were analysed using an Accuri C6 flow cytometer (BD Biosciences).

### Isolated perfused rat heart

Male Sprague Dawley rats (300–350 g) were anaesthetized with sodium pentobarbital (60 mg/kg) i.p. The heart was excised, cannulated via the aorta, and retrogradely perfused using a Langendorff apparatus, with a modified HEPES-based Tyrode’s solution because Krebs buffer was found to contain larger numbers of nano-sized calcium precipitates that interfered with NTA quantification of exosomes. The buffer consisted of: 25 mM HEPES, 119 mM NaCl, 4.8 mM KCl, 1.2 mM CaCl_2_, 2 mM MgCl_2_, 11 mM glucose adjusted to pH to 7.4 with NaOH, gassed with O_2_ and maintained at 37 °C. For IPC, the hearts were made globally ischaemic by stopping flow into the heart for 5 min, then reperfused. The perfusate was collected for 10 min prior to, and 10 min after IPC. EVs were isolated by differential centrifugation as described, and analysed by NTA.

### Statistical Analysis

The N number indicates independent biological replicates. Data is shown as mean ± SEM. Where samples were taken from the same heart before and after IPC, statistical comparison was by Student’s paired T-test. For multiple comparisons, one-way ANOVA was followed by post-test analysis using the Tukey test for multiple comparisons. For the analysis of time-course release of exosomes, repeated measures ANOVA was used with Bonferroni post-test. P < 0.05 was considered significant. *P < 0.05; **P < 0.01, ***P < 0.001.

## Results

**Figure 1:**
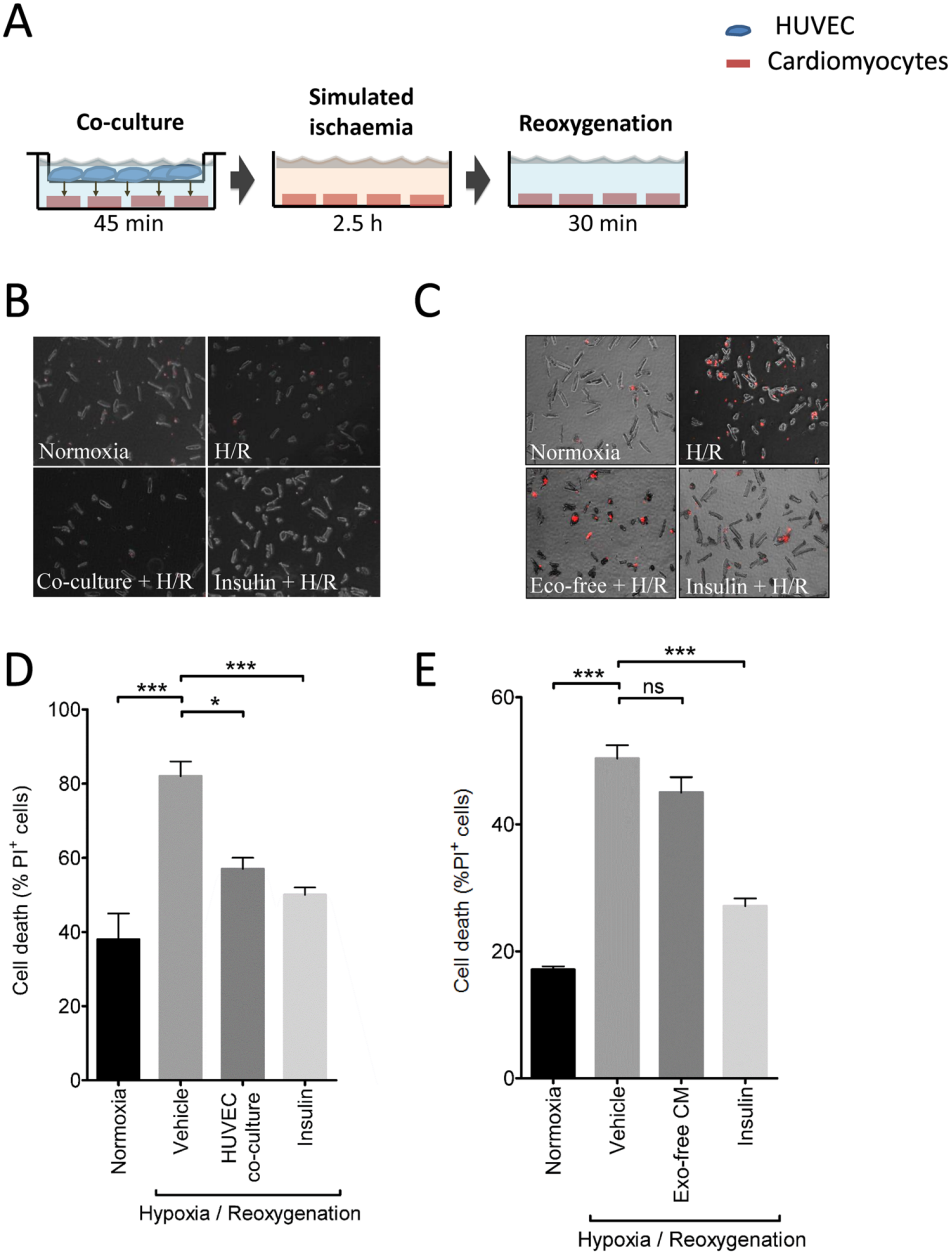
HUVEC release cardioprotective factors. (**A**) Experimental protocol for the co-culture of HUVEC with adult rat cardiomyocytes using a trans-well system, followed by hypoxia and reoxygenation (H/R). Separate wells of cardiomyocytes were maintained in normoxic medium. Insulin pre-treatment was used as a positive control for cardioprotection. (**B**,**C**) Representative images of cardiomyocytes stained with propidium iodide (PI) at the end of the H/R protocol. Fluorescent images (PI staining in red) were overlaid with phase contrast images of cells. (**D**,**E**) Percentage of dead cells after normoxic culture, after H/R, after HUVEC co-culture (D, N = 5), or after addition of exosome-free conditioned medium (“exo-free CM”) (E, N = 3). *P < 0.05, ***P < 0.001.

To investigate whether endothelial cells release soluble factors that can deliver cardioprotective signals to cardiomyocytes, adult rat cardiomyocytes were co-cultured with HUVEC endothelial cells, separated by a 0.4 μm filter membrane in a trans-well system. After 45 min exposure, the insert containing the endothelial cells was removed, and the cardiomyocytes were incubated in a buffer simulating ischaemia and placed in a hypoxic chamber for 2.5 h followed by re-oxygenation for 30 min (H/R) (Fig. 1A). Co-culturing HUVEC and cardiomyocytes for 45 min prior to H/R significantly reduced the percentage of cell death from 80 ± 11% to 51 ± 4% (P < 0.05; N = 5) (Fig. 1B,D). The degree of protection was similar to a positive control in which the cardioprotective RISK pathway^5^ was activated using insulin (Fig. 1B,D). HUVEC-conditioned medium was collected, and ultracentrifuged at 100,000 h for 20 h to remove all extracellular vesicles (EVs). When this medium lacking EVs was added to cardiomyocytes prior to hypoxia and reoxygenation it did not provide any protection (Fig. 1C,E), suggesting that HUVEC release cardioprotective factors, which we hypothesized to be EVs or more specifically, exosomes.

**Figure 2:**
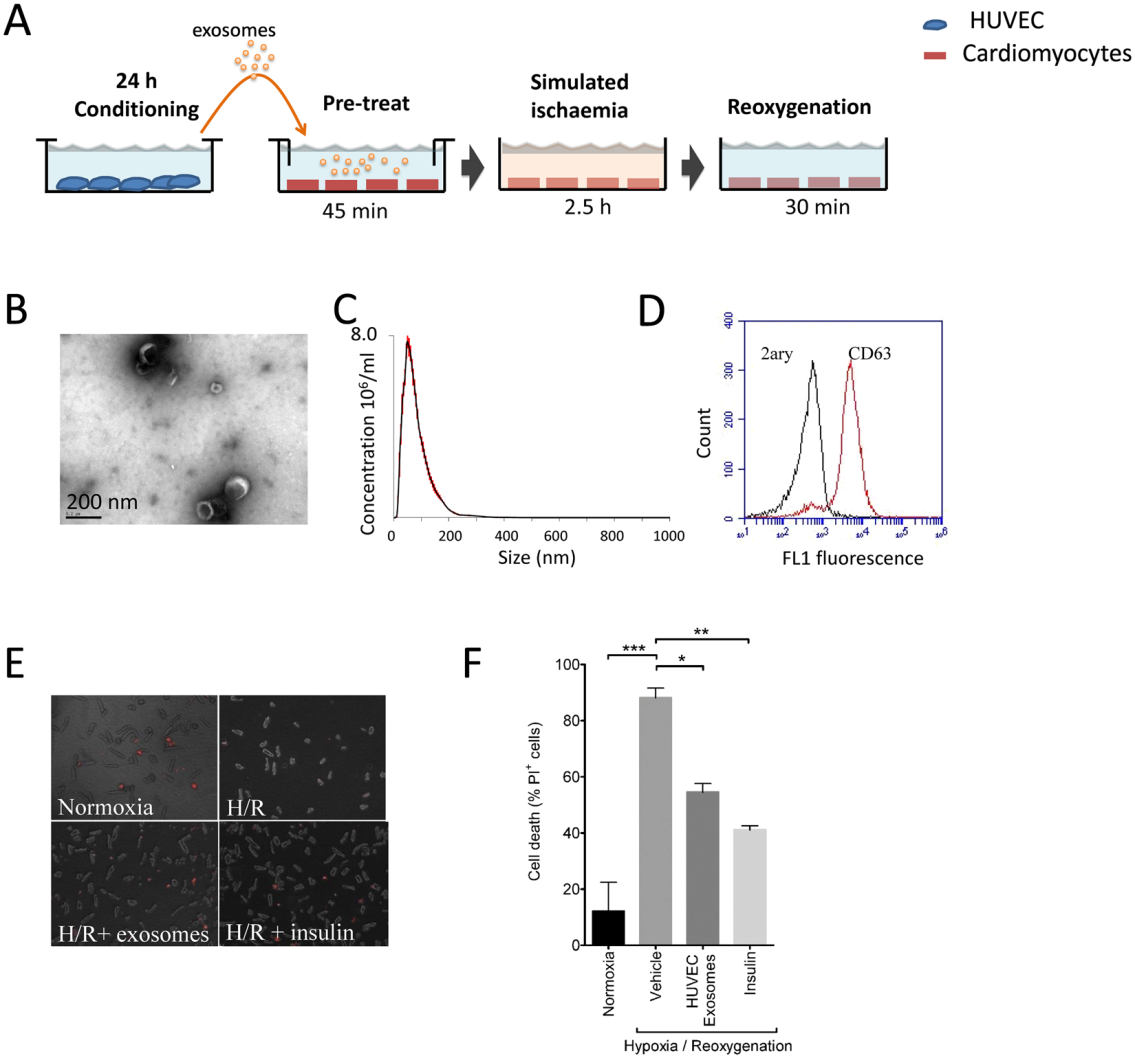
HUVEC release cardioprotective exosomes. (**A**) Experimental protocol in which cardiomyocytes were pre-treated with HUVEC exosomes before H/R. Separate wells of cardiomyocytes were maintained in normoxic medium. Insulin pre-treatment was used as a positive control for cardioprotection. (**B**) Electron micrograph of purified exosomes from HUVEC cells. (**C**) Representative size and concentration distribution of HUVEC exosomes purified from HUVEC cells after 24 h culture, determined by nanoparticle tracking analysis. (**D**) Flow cytometric analysis exosome-coated microspheres confirmed the presence of exosomal marker CD63. (**E**,**F**) Percentage of dead cells after normoxic culture, after H/R, after exposure to HUVEC exosomes, or insulin (N = 3). *P < 0.05, **P < 0.01, ***P < 0.001.

EVs were isolated from HUVEC-conditioned medium using a standard method of differential ultracentrifugation^20^, and characterized to confirm their identity as exosomes. Using transmission electron microscopy (TEM) we observed the typical “cup-shaped” exosomes with a diameter <200 nm (Fig. 2B). To further characterize and quantify exosomes, we used nanoparticle tracking analysis (NTA), which showed that the distribution of vesicle diameters centred around 50–150 nm, as expected for exosomes (Fig. 2C). The modal size was 82 ± 6.3 nm, and the average concentration was 3.4 × 10^10^ ± 1.4 × 10^10^/ml. We bound the vesicles to 4 µm beads, labelled them to antibodies, and performed flow cytometric analysis to demonstrate the presence of the exosome marker proteins CD63 (a molecule of the tetraspanin family) (Fig. 2D). Together, these data confirmed the vesicles as exosomes. Next, we pre-incubated cardiomyocytes with 10^8^/ml HUVEC exosomes, prior to subjecting them to sIR. Exosome treatment reduced the percentage of cell death from 88 ± 4% to 55 ± 3% (P < 0.05; N = 3) (Fig. 2E,F).

**Figure 3:**
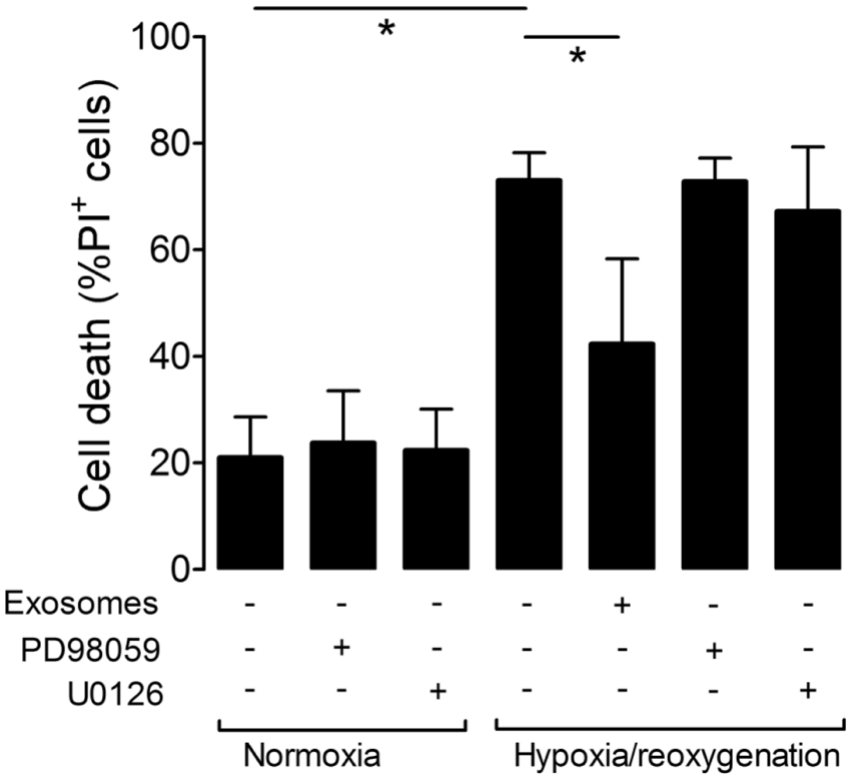
HUVEC exosomes protect cardiomyocytes via the ERK1/2 signalling pathway. The ERK1/2 inhibitors U0126 and PD98059 prevented protection of cardiomyocytes by HUVEC exosomes (N = 3; *P < 0.05).

We previously showed that treatment of cardiomyocytes with HUVEC exosomes stimulates the phosphorylation of ERK1/2, but not Akt^17^, although we did not confirm that ERK1/2 was involved in the cardioprotective pathway of HUVEC exosomes. Here, therefore, we investigated this using two different inhibitors of ERK1/2 signalling, 10 μM U0126 or 50 μM PD98059. Pre-treatment of cardiomyocytes with either drug eliminated cardioprotection by HUVEC exosomes (Fig. 3).

**Figure 4:**
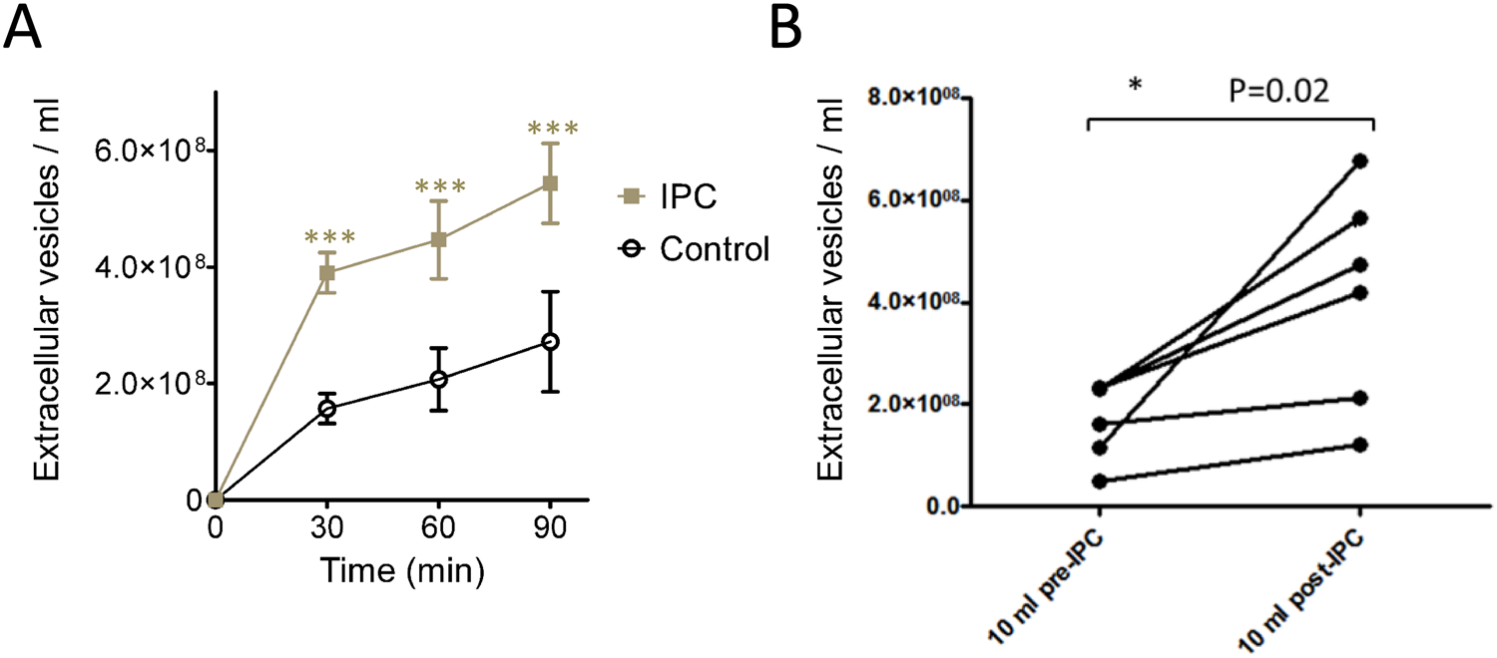
Ischaemic preconditioning (IPC) increases the release of EVs. (**A**) The increase in exosome concentration in HUVEC culture medium over time (N = 3). (**B**) Ischaemic preconditioning of Langendorff perfused hearts significantly increase the rate of exosome release measured after 10 min (N = 6).

We investigated whether ischaemic preconditioning would stimulate exosome release. Using NTA, the basal rate of exosome release from HUVEC over 24 h was calculated to be ~37 exosomes per cell per min (Fig. 4A). After *in vitro* preconditioning with 30 min transient exposure to simulated ischaemia, the rate of exosome release approximately doubled (N = 3, P < 0.001) (Fig. 4A). To confirm this also occurs in an intact organ, we subjected an isolated, Langendorff perfused rat heart to IPC, and isolated exosomes from the perfusate. Again, and despite some heterogeneity between hearts, we saw a significant increase in the number of exosomes released. Overall, the average concentration of exosomes increased by 2.8 ± 1.0 fold (from 1.4 ± 0.4 × 10^8^/ml to 3.2 ± 0.4 × 10^8^/ml) (N = 6, P = 0.02) (Fig. 4B). Exosome size did not change (99 ± 5 nm before and 91 ± 6 nm after).

**Figure 5:**
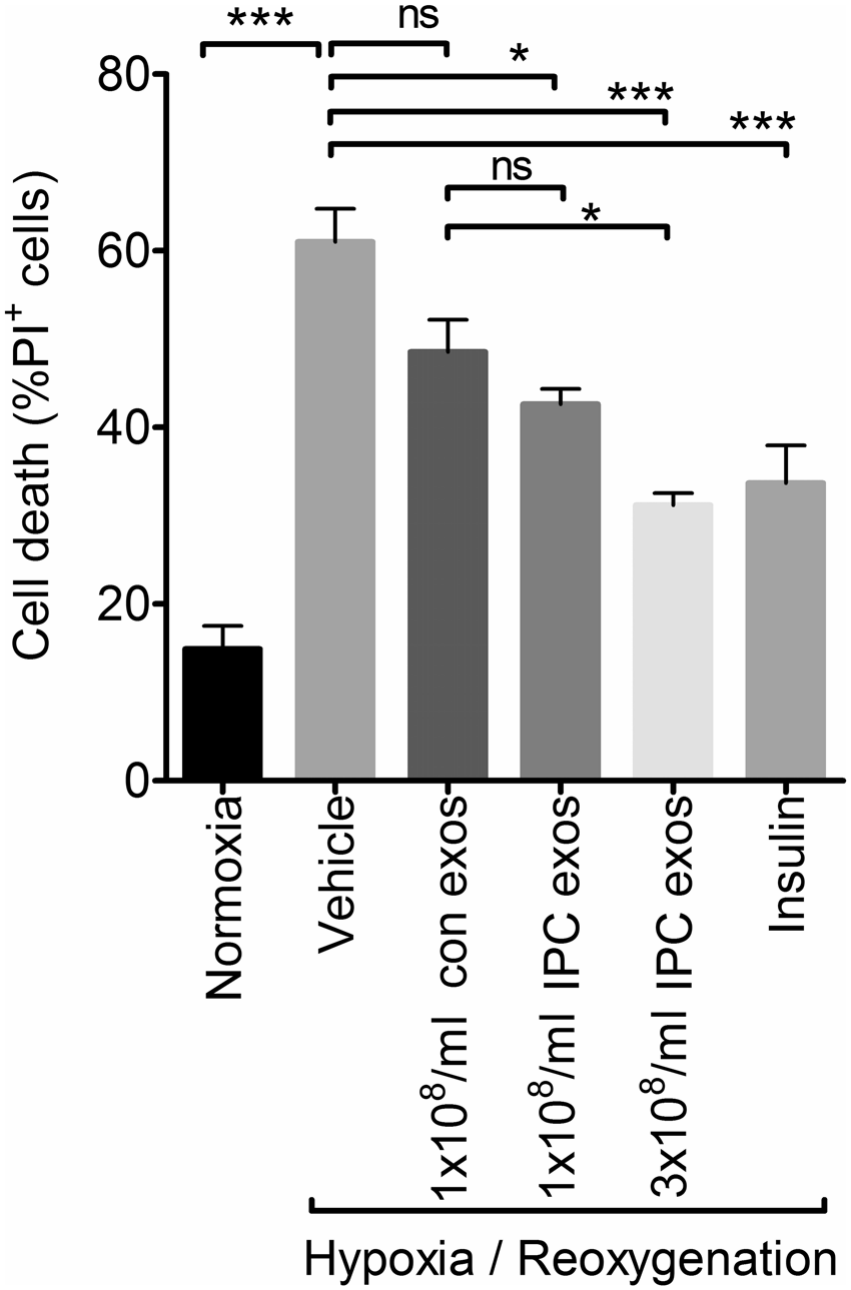
The effect of ischaemic preconditioning on protection by endothelial exosomes. Cardiomyocytes were pre-treated with the indicated concentration of exosomes from control HUVEC (con exos) or those that had been subject to a preconditioning stimulus (IPC exos), before analysis of cell death after H/R. Insulin pre-treatment was used as a positive control for cardioprotection. (N = 3; *P < 0.05, ***P < 0.001).

Finally, we investigated whether exosomes produced by HUVEC after preconditioning were more protective than those from control cells. In this set of experiments, treatment with 10^8^/ml exosomes from either control or preconditioned cells slightly protected cardiomyocytes against H/R, although only the preconditioned exosomes caused a significant reduction (P < 0.05) (Fig. 5). When added at equivalent concentrations, the difference between control and preconditioned exosomes was not significant (Fig. 5). On the other hand, when the concentration of preconditioned exosomes was increased by a factor of 3, to reflect the increased number of exosomes produced after preconditioning, a significantly greater degree of protection was observed compared to control exosomes (Fig. 5).

## Discussion

Exosomes and other EVs are released into the circulation by various cells including platelets, erythrocytes, lymphocytes and endothelial cells. We were interested in whether exosomes released from endothelial cells have cardioprotective properties. Using an *in vitro* model of adult rat cardiomyocytes, we showed that cultured endothelial cells release certain substances that protect the cardiomyocytes against subsequent hypoxia and reoxygenation. Removal of EVs from the HUVEC-conditioned medium eliminated protection, whereas the EVs purified from HUVEC-conditioned medium were shown to be protective. Given the typical size and shape of the EVs, and expression of the tetraspanin CD63 commonly used as a marker of exosomes, these EVs were designated as exosomes, although we did not formally exclude the presence of low quantities of other EVs. Pre-treatment of cardiomyocytes with two different inhibitors of the MEK1/2 – ERK1/2 kinase pathway eliminated cardioprotection by HUVEC exosomes. Preconditioning rapidly increased the number of exosomes released by isolated, perfused rat hearts by ~3-fold. Although preconditioning did not affect the cardioprotective activity of exosomes when added at equimolar concentrations, a ~3-fold increase in concentration resulted in a significantly greater degree of protection. The results of this study lead us to conclude that exosomes released from endothelial cells can confer resistance to IR injury in cardiomyocytes via the activation of the ERK1/2 MAPK signalling pathway, and may contribute to the mechanism of myocardial IPC.

Cardiomyocytes in the myocardium are outnumbered by approximately 3:1 by endothelial cell in overlying capillaries, arterioles and larger vessels^18^. These endothelial cells can modulate the activity of cardiomyocytes. For example, endothelial nitric oxide can influence cardiac substrate utilization and contraction^19^. This suggests the interesting possibility that endothelium could communicate to neighbouring cardiomyocytes via exosomes. In a recent study in mice, EVs and exosomes were isolated from the interstitium of minced cardiac tissue, and found to be significantly increased in number after 15 h coronary occlusion^21^. These EVs were shown to modulate the inflammatory response although this effect appeared to be limited to the larger EVs^21^. For our studies, we used HUVEC as a convenient *in vitro* model of endothelial cells. However, it would be interesting to perform similar experiments using primary endothelial cells isolated from an adult vascular bed, or those isolated from an arterial vessel.

A limitation of all such *in vitro* studies is that the exosomes released from cells in culture may not completely recapitulate the release of exosomes *in vivo*. However, it is currently technically challenging to investigate the role of exosomes from an individual cell type in an *in vivo* setting. An aspect of our study that we believe is important is the use of adult cardiomyocytes. These have very distinct properties from immature neonatal cardiomyocytes, and evidence to date suggests they are much less capable of endocytosing exosomes than other cell types^16,22,23^. It should also be noted that the fluorescent lipophilic dyes that are commonly used to detect uptake are highly subject to artefact^24^. In any case, the deficiency in exosome endocytosis by cardiomyocytes, in combination with the fact that protection is achieved after only 45 min exosome treatment, would suggest that transfer of miRNA is not responsible for the protection observed. However, it should be recognized that a number of previous studies have implicated miRNA in longer-term cardioprotection mediated by exosomes (reviewed in^13,25,26^). For example, EVs produced by human cardiac progenitor cells, which are able to improve cardiac contractile function in a permanent ligation rat model of acute MI, were found to contain high levels of miR-210, miR-132, miR-146a-3p^27^. Furthermore, miR-144 was found to be increased in circulating exosomes after RIPC, potentially mediating RIPC-induced cardioprotection^28^. Recently, exosomal miR-21a-5p has been shown to mediate cardioprotection by exosomes produced by mesenchymal stem cells that were administered 24 h prior to IR^29^. Exosomes are believed to protect the sequestered proteins and RNA from degradation, facilitating their delivery to recipient cells. In this regard, exosomes are an active component of the paracrine secretion by cardiac (and other) cells, and are therefore attracting much interest, as recently rewiewed^11–13^.

To directly address the mechanism of protection we used two distinct inhibitors of ERK1/2 and showed that they blocked protection by HUVEC exosomes. HUVEC-conditioned medium has also been shown to protect human intestinal cells (CaCo-2) against IR injury via ERK1/2, although the factor responsible was not identified^30^. Previously, we found that cardioprotection by exosomes isolated from blood plasma is dependent on ERK1/2 signalling^16^. This kinase was phosphorylated in response to activation of the TLR4 receptor pathway by HSP70 on the exosomal surface^16^. Interestingly, however, there does not appear to be any HSP70 on the surface of HUVEC exosomes^17^. Thus, the mechanism by which HUVEC exosomes activate ERK1/2 in cardiomyocytes remains unknown and is the subject of future studies.

The involvement of endothelium in IPC has important consequences, because many co-morbidities commonly present in cardiac patients such as diabetes and age impair endothelial function. Hyperglycaemia has been shown to impair the production of nitric oxide and interfere with transmission of the cardioprotective signal from preconditioned endothelial cells^31^. Furthermore, we showed that hyperglycaemia also impairs the production of cardioprotective exosomes^17^. Furthermore, exosomes isolated from diabetic rats or humans exhibited impaired cardioprotection^17^, although as stated above, it is currently challenging to determine the cellular origin of the protective exosomes contained within plasma. Most studies of exosomes and cardioprotection have used exosomes isolated from stem or progenitor cells, and whether exosomes from other cell types are able to stimulate protection has not been comprehensively investigated. The exception is fibroblast exosomes, which appear to lack activity in most cardioprotective studies – at least those that are isolated from primary fibroblasts^13^.

Preconditioned hearts release exosomes that are cardioprotective when transferred to naïve hearts, suggesting that they may also contribute to the mechanism of remote preconditioning^9^. We saw a consistent, though somewhat variable, increase in the number of exosomes after preconditioning isolated hearts. We did not determine the cell type of origin of these exosomes, but a recent study saw no increase in exosome numbers after *in vitro* preconditioning of cardiomyocytes or fibroblasts^8^, which indirectly suggests that the cardiac exosomes may originate from the endothelium. We previously detected a small, but significant, increase in plasma exosomes after RIPC, although when these were used to treat cardiomyocytes *in vitro*, RIPC exosomes were not significantly more protective that control exosomes (at equivalent concentrations)^16^. As it is not yet possible to specifically inhibit exosome production *in vivo*, let alone from a specific cell type, we are unable to definitively determine the role of exosomes in RIPC.

In future studies, it will be important to establish the extent to which cardiac microvascular endothelial cells communicate to cardiomyocyte via exosomes, and can influence their metabolism, activity, and potentially resistance to IR injury. It will also be important to determine the active protective component within the exosomes, which it may then be possible to isolate and develop as a cardioprotective agent. On the other hand, the exosome vehicle may be a necessary component for delivery of the cardioprotective reagent, in which case, improved preparative methods will be necessary in order to develop exosomes as a potential cardio-therapeutic agent.

## Data Availability

The datasets generated during and/or analysed during the current study are available from the corresponding author on reasonable request.
